# The Necessity for Yet Further Extension of Clinics

**Published:** 1920-02-28

**Authors:** 


### OBSTETRIC TEACHING.

### The Necessity for Yet Further Extension of Clinics.

Only a few days ago a singularly unfortunate obstetric case was admitted as an emergency admission at one of our great London Hospitals. The patient, a young woman of about thirty years, had been treated at her house by a practitioner, who had done his best to extract a fœtal transverse presentation but without success: much trauma and hæmorrhage had attended his efforts, and the patient was admitted to the hospital in an exceedingly exsanguine and shocked condition. An immediate operation was performed, the transverse presentation being extracted by means of decapitation; but alas! in spite of the best medical attention the patient gradually became more exhausted, and finally expired a few hours after admission.

In this case the medical treatment was magnificent, the practitioner and the hospital specialists both did their work with skill and the utmost promptitude, and yet, in spite of all their exertions, we have to record the sad death of both mother and child. Now such things need not be, for if only this case had been examined ante-natally prophylactic measures would have been instituted and two lives might have been saved.

We suppose that one of the commonest causes of this transverse malpresentation is multiparity; the fœtus would have been lying in its malposition for some weeks before delivery, a simple examination would have revealed this fact, and an external version would have at once rectified the position of the child. Or again, the transverse presentation may have been caused by a placenta prævia, a pelvic deformity or obstruction, the existence of hydramnios, or a deformity on the part of the fœtus; but whatever the cause, if the case were examined ante-natally the life of the mother could always be saved, and in most instances that of the child as well.

We have only mentioned this extreme case as an actual fact, so as to impress the immediate need for the institution of clinics. The uses of such clinics would be legion, and they would undoubtedly save much trouble to expectant mothers, and bring about a very material decrease in the appalling infant mortality which exists in this country.

### The Use of Clinics.

Of paramount importance is the recognition of malpresentations of the fœtus, and great disparity in size between the presenting fœtal extremity and the osseus pelvic girdle: either of these complications may well bring about very difficult or fatal labours. In the former instance these can be rectified simply and with success, and in the latter the horrors of obstructed labour can be avoided by a suitable induction of labour when the infant is viable, or else the performance of Cæsarian section at term.

The condition of placenta prævia is unfortunately not a rare occurrence; this abnormality is one of the chief causes of ante-partum hæmorrhage, and is associated with a very great mortality. Such placentæ can be diagnosed ante-natally and suitable treatment instituted to prevent future trouble.

Early and prompt treatment of the toxæmias of pregnancy is essential for cure, and their earliest symptoms can best be elicited by a systematic course of ante-natal examinations. Thus eclampsia, hyperemesis gravidarum, and the pyelitis of pregnancy will all give the obstetrician certain premonitory signs, which, if only recognised, will lead to their immediate treatment and subsequent possible cure.

### The Treatment of Syphilis.

The ravages of syphilis in relation to pregnancy are too well known to deserve minute description; suffice it to state that a very considerable percentage of the existing fœtal mortality is due to this disease, and even in those children who, having escaped miscarriage and premature or still-birth, there remains the melancholy prognosis of congenital syphilis. If only the disease is recognised in good time, the modern intensive treatment by means of salvarsan and mercury injections may be instituted and a negative Wassermann reaction secured, at any rate in the mother. In some cases the life of the fœtus in utero will also be saved, and from recent statistics the perfection of modern technique and dosage of ante-syphilis remedies is showing a definite increase in the births of healthy full-term babies without any congenital venereal taint. Of course, in some cases treatment of a pregnant patient with a positive Wassermann reaction is attended by miscarriage; but even if this be the case it matters little, as a miscarriage is such a frequent sequel to a syphilis pregnancy, and this event is quite overshadowed by the great success achieved if the mother is cured of her disease, and is thus rendered potentially fertile to secure the addition of healthy babies to her family.

We may also mention that the secondary stage of syphilis has a very detrimental effect upon the puerperium, and in the increase of maternal mortality from sapræmia, septicæmia, and other septic conditions of the uterus, these being associated with the profuse and foul discharge so often associated with vulval condylomata. The efficient treatment of syphilis in pregnancy is exceedingly difficult without an adequate system of ante-natal examination, for although some stages of syphilis will attract attention by cutaneous manifestation, the disease is just as often hidden and only recognisable to the trained eye of the clinician.

### The Educational Value of Clinics.

Of especial value to the primipara and indeed to all expectant mothers would be a course of ante-natal instruction with at least a few words of advice as to the conduct of life during the months of pregnancy. Improvements in personal hygiene being taught, proper exercise indicated, the diet and the bowels regulated, the teeth properly cared for, and a suitable local treatment of the nipples and

**Improved Obstetric Teaching**—(*continued*).

breasts instituted. The tendency towards abortion from accidental causes would thus be lessened, and the frequence of "milk fever" or mastitis diminished. The mental attitude of the expectant mother should also be suitably moulded, so that she is reassured as to her parturition and is taught to regard the process as a purely physiological function: thus the "dread of pregnancy" being diminished, deliberate prevention of conception would in some measure be discouraged.

The Suitable Provision for "Lying in."

In this provision we state frankly that we are striving for the ideal, which would consist of public "lying-in" wards, numerous enough to secure accommodation for the accouchements of our poorer population. Such accommodation is of paramount importance in the case of difficult or complicated labours, but even in the case of the most normal labours definite advantage would accrue to mother, child, and State. For we must all realize that the greater bulk of our population is born among surroundings which are just about as unsuitable as could be imagined. Contrast only for a moment the obvious advantage of a properly attended labour in a clean sanitary lying-in ward as opposed to the badly attended labour in an often filthy room, ill-lit by a miserable gas jet, fearsomely hot in winter or summer, and in a chamber which may often be the sole abode of the family into which another soul is to be born.

Exception may be taken to the term "badly attended labour," but we use this phrase after deliberation, for we have formed the opinion that such is really the case, and this is chiefly due to the uncertain time at which parturition may take place. The average medical attendant in our poorer neighbourhoods is an exceedingly busy man, and however good his intentions he needs must often arrive after the baby is born or not have sufficient time to perform all the duties which he would be able under the ideal conditions of a system of public "lying-in" wards.

We do not suggest for one moment that every woman should have her child in a public "lying-in" ward, but only amongst the poorer classes of our populations, where ideal provisions are remarkable for their entire absence.

Any schemes of ante-natal clinics would lose much of their potential value if their benefits ceased with the birth of the child. Many and many a healthy child is born only to succumb to faulty hygiene during the first year of its life, and such infant mortality is a blot upon the fair fame of medicine, and a disgrace to the Government of this country.

It is common knowledge to both the medical profession and the laity that such mortality is in a great measure preventable.

